# The Evaluation of the Possibilities of Using PLGA Co-Polymer and Its Composites with Carbon Fibers or Hydroxyapatite in the Bone Tissue Regeneration Process – *in Vitro* and *in Vivo* Examinations

**DOI:** 10.3390/ijms10073224

**Published:** 2009-07-15

**Authors:** Magdalena Cieślik, Anna Mertas, Anna Morawska-Chochólł, Daniel Sabat, Rajmund Orlicki, Aleksander Owczarek, Wojciech Król, Tadeusz Cieślik

**Affiliations:** 1 Chair and Department of Stomatological Material Science, Pl. Akademicki 17, 41 907 Bytom, Medical University of Silesia, Katowice, Poland; E-Mail: materialoznawstwo@wp.pl (R.O.); 2 Chair and Department of Microbiology and Immunology, Jordana 19, 41 808 Zabrze, Medical University of Silesia, Katowice, Poland; E-Mails: amertas@sum.edu.pl (A.M.); wkrol@sum.edu.pl (W.K.); 3 Department of Biomaterials, Faculty of Materials Science and Ceramics, Al. Mickiewicza 30, 30 059 Cracow, AGH University of Science and Technology, Cracow, Poland; E-Mail: anmore@op.pl (A.M.-C.); 4 Department and Institute of Pathomorphology, 3maja 13-15, 41 800 Zabrze, Medical University of Silesia, Poland; E-Mail: d.sabat@wp.pl (D.S.); 5 Division of Statistics, Ostrogórska 30, 95 010 Sosnowiec, Medical University of Silesia, Katowice, Poland; E-Mail: aleksander.owczarek@gmail.com (A.O.); 6 Department and Clinic of Maxillofacial Surgery, Francuska 20/24, 40 027 Katowice, Medical University of Silesia, Katowice, Poland; E-Mail: tcieslik@sum.edu.pl (T.C.)

**Keywords:** composites, PLGA co-polymer, carbon fibers, hydroxyapatite, human osteoblast-like cells, cytotoxicity, *in vivo* experiments on animals, biocompatibility

## Abstract

Synthetic polymers belonging to the aliphatic polyester group have become highly promising biomaterials for reconstructive medicine. The purpose of the present work is a biological evaluation of lactide-glycolide co-polymer (PLGA) and its composites with carbon fibers (PLGA+CF) or hydroxyapatite (PLGA+HA). The cytotoxicity of the evaluated materials towards hFOB 1.19 human osteoblast-like cells was assessed. Moreover, during the one-year contact of the assessed materials with living osseous tissue, the progress of bone formation was analyzed and the accompanying process of the materials’ degradation was evaluated. The materials under evaluation proved to be biocompatible.

## 1. Introduction

The growth of material engineering dealing with the development of new, biocompatible implant materials is strongly motivated by the difficulties connected with obtaining an adequate number of organs for transplantation, the possibility of their rejection by the recipient or the additional difficulties experienced by the patient connected with the operation. The demand is growing for new, innovative material solutions which are characterized by better physico-mechanical qualities or general biofunctionality [1]. The progress in research techniques offers the possibility of developing new materials with improved biological compatibility as well as verifying the existing solutions and excluding the biomaterials which may have a negative influence upon the human body [2,3].

Bioresorbable lactide-glycolide co-polymer (PLGA) is arousing a great interest in the area of biomedical sciences. Many years’ research followed by scientific works have confirmed that it is possible to use PLGAs in medical specialties such as tissue and genetic engineering, pharmacology, surgery or stomatology [4–9]. They are mainly used to restore soft tissue defects, as medicine carriers their controlled release in the living body or else as a cell basis [10–13]. The reason why they are employed in such ways is their low endurance parameters, which limit their usefulness to elements which do not transfer great mechanical loads [14–16]. Using them as supporting or stabilizing elements is possible after creating composites based on them.

PLGA co-polymer blends with different kinds of fillers of natural and synthetic origin. Most frequently, carbon fibers or fibers made of the same material as the polymer matrix (self-reinforced materials) are used for this purpose [14,17,18]. Moreover they are blended with ceramics based on calcium phosphates (hydroxyapatite, tricalcium phosphate), which influences the bioactivity of the composite, or bio-glass [19–21]. The modifying phase present in the polymer matrix has an influence on the mechanical properties of the composite, cell response and the process of its degradation.

The time and progress of PLGA-based composites’ degradation processes after performing their function in the body are very significant aspects determining their usefulness. The factors determining this phenomenon are, among others, the degree of their crystality, molar mass, porosity, pH or the environment temperature [10,14,15,17,22]. Additionally, the proportion of glycolide to lactide, as well as the manner of making the co-polymer are of great significance. Using biocompatible polymerization initiators (such as zirconium, zinc, calcium or iron compounds) during the synthesis process increases its degree of inertness [23,24]. Apart from that, the kind of living tissue adjacent to the composite has a great influence on the progress of degradation processes.

Biocompatible carbon fibers play an important role in composite creation. Their presence in polymers allows for development of materials characterized by an advantageous endurance-elasticity relationship in relation to their density; moreover, there are permeable for X-rays. Additionally, the use of carbon fibers to create fibrous, three-dimensional cell bases to be used, among other applications, in bone defect restoration is a great challenge for tissue engineering [14]. They imitate the natural structure and biological functions of the intercellular substance. They also often have the three-dimensional, porous structure with a pore size optimal for the growth, proliferation or adhesion of the particular cell types involved [25].

Composites containing hydroxyapatite have a different set of features. This filler is characterized by the highest biocompatibility and bioactivity of all known implant materials. Thanks to its osteoconductive and osteoinductive properties, implants containing it as an ingredient can fasten together directly with the bone (biological binding) [19,20,26]. Moreover, it has a positive influence upon bone healing and bone restoration because of its ability to initiate and stimulate the processes involved. It plays a particularly important role when long-lasting bone restructuring processes are required [26,27].

The present work attempts to discuss and evaluate the biological properties of two new composites based on lactide-glycolide co-polymer (PLGA) in *in vitro* and *in vivo* examinations. The aim of combining it with carbon fibers (PLGA+CF) or hydroxyapatite (PLGA+HA) was to develop modern biomaterials supporting the healing process and bone tissue regeneration.

## 2. Results and Discussion

### 2.1. In Vitro Examination on Human Cells

The results of microbiological examinations determining the percentage value of the cytotoxicity (LDH test) of PLGA co-polymer and its composites: PLGA+CF and PLGA+HA on hFOB 1.19 human osteoblast-like cells are presented in Table 1. The data show that in all the measurement periods the cytotoxicity values of the materials under evaluation were within the allowed limits [28]. Its highest value was recorded after 24 hours for PLGA+HA composite, and its lowest value was for PLGA+CF composite. The presence of biologically active hydroxyapatite nanoparticles in this material probably accelerated the stimulation of the adjacent cells. On the other hand, carbon fibers without such properties acted on the same osteoblasts in a more neutral way. More intensive cell reaction on the “foreign body” (PLGA, PLGA+CF) took place after 48 hours – resulting in the highest cytotoxicity values. Additionally, the cumulative toxic influence of all the materials upon the osteoblasts used in the first two periods (24 h, 48 h) could be caused by the inevitable chemical reactions characteristic of the test employed [12,29].

Comparing the results of this kind of examinations with other researchers’ work it should be kept in mind that many factors influence the cells’ final response in the contact with polymer biomaterial; these factors may be of a chemical, physical, technological or biological nature. The kind and the origin of the cells used are of particular importance, as well as the way they are put in contact with the samples of the examined material [12,13,21,25]. With regards the performance of the cells, their activity is influenced by the kind of the polymer base, which can vary depending on the processing method, the kind of modifying phase, the size of the filler particles, surface topography, porosity, resorption degree or the molecular weight [30].

### 2.2. In Vivo Examinations on Animals

**Figure 1.**
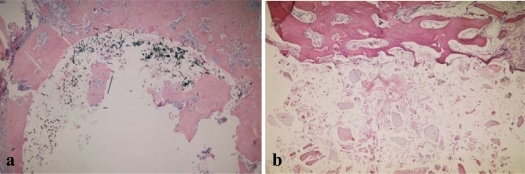
Histopathological picture (24^th^ week – H.E. tinting, magnification 50x) – bone canal covered with mature bone tissue without traces of osteoblastic activity: PLGA+CF composite (a), PLGA+HA composite (b).

After the 21^st^ day the histopathological pictures demonstrated in both composites the presence of bone trabeculae without traces of osteoblastic activity, adjacent to the connective tissue covering the bone canal. It the same time, the bone trabeculae demonstrated traces of vivid cellular activity in the presence of PLGA co-polymer – numerous osteoblasts could be seen on their surface. After 6 weeks of observations, only scarce, single bone trabeculae bordered by osteoblasts could be seen in both the PLGA+CF and PLGA+HA group, whereas in the PLGA group vivid osteoblastic activity could still be observed and only the edges of the defect were covered with mature bone tissue. In the case of both composites, as early as 12 weeks after the examinations, the bone canal was filled with mature bone tissue without traces of bone-forming activity, which expanded intensively in the further examination periods (Figure 1). Analyzing the co-polymer itself, it was concluded that complete filling and healing of the bone defect took place after 48 weeks of examinations. The process was similar in the case of the control group, where the total disappearance of osteoblastic cells could be observed only after 24 weeks of the examinations.

In the case of both composites, “around-foreign-body”- type cocci were observed until the 6^th^ week of the examination. They were placed around single carbon fibers located outside the connective tissue capsule (in PLGA+CF) and around hydroxyapatite nenoparticles set among the expanding fibrous tissue strands (in PLGA+HA). It can be presumed that the fillers detached from the transplants undergoing degradation were dislocated and disintegrated into smaller parts. This caused an increased cellular defense reaction [31].

Moreover, in the case of the composites, faster disintegration and resorption of the polymer matrix took place, in comparison with the co-polymer alone. After 24 weeks of the examination only in the PLGA group were there numerous co-polymer fragments among the strands of fibrous tissue.

The presence of fillers in the composites’ warp had a major influence upon the process of the composites’ degradation and faster growth of bone tissue in the defect where the composites were present [10,14,15,19,20]. The reason for that was the fact that at the junction points, or at the phase borders of both carbon fibers and hydroxyapatite nanoparticles, the implant disintegration process was initiated. The lodgements of the polymer matrix dispersed in the bone tissue interlacing them had a greater contact with the biological environment supporting a faster resorption process [14]. What followed was a more intensive bone-formation process, since the newly-formed bone tissue had a better possibility of penetrating into the implant. Analyzing the results of histopathological examinations one can also presume that the fillers detached from the polymer matrix became the points of osteogenesis initiation. It is particularly justified in the case of biologically active ceramics which has the property of arousing bone-forming cells (osteoblasts) [1,2,16].

During immunohistochemical examinations, positive reactions against osteonectin and osteopontin became evident in the form of brown-tinted cell cytoplasm or matrix. In the first case, positive reactions were observed in the osteoblasts of the bone trabeculae, tooth odontoblasts, blood vessels endothelia and bone marrow megacariocytes; whereas in the other case – in the extracellular matrix of the new bone tissue and also in osteoblasts and odontoblasts (Table 2).

Particularly strong reactions were visible in loosely located osteoblasts, on the surface of bone trabeculae and in their matrix for PLGA co-polymer and its composite with HA. A less intensive mineralization process and osteoblasts activity was observed in the case of the PLGA+CF composite. It was probably caused by the presence of carbon fibers which have a weaker stimulating effect on cells [18,25]. It can also be assumed that surface antigens could have been damaged during the tissue decalcification process.

## 3. Experimental Section

### 3.1. General

The material used in the present work was lactide-glycolide co-polymer (PLGA): 82% lactide and 18% glycolide produced in the Polymer and Carbon Materials Centre of the PAN (Polish Science Institute) in Zabrze. The synthesis was carried out by ring opening using a non-toxic initiator-zirconium acetylacetonate [23,24]. The co-polymer was characterized by medium particle weight Mn=75 kDa and the polydispersion index Mn/Mw=2.1. The PLGA+CF composite was produced by adding to the co-polymer 15 wt % short carbon fibers (CF): FT 300B (Torayca, Japan; fibre diameter d=7 µm, density ρ=1.76 g/cm^3^, tensile strenght σr=3.2 GPa, Young module E=235 GPa). Before mixing carbon fibers with the dissolved polymer, they were processed thermally in order to remove preparation (400 °C, 30 min.). In the case of PLGA+HA hydroxyapatite (HA) nanoparticles of natural origin (bovine bone) were used in the amount of 15 wt % (surface unfolding p=79.7 m^2^/g, density ρ=3.16 g/cm^3^) [32].

### 3.2. Samples

For *in vitro* examinations discs of 16 mm diameter and 1 mm thickness were used; they were cut from membranes obtained by pouring co-polymer and its composites from CH_2_Cl_2_ solution on platters. In order to improve the homogeneity of the PLGA+HA composite, before adding the hydroxyapatite nanoparticles to the co-polymer dissolved in CH_2_Cl_2_, the nanoparticles were moistened in the same solution. To combine both components they were mixed on a magnetic mixer and broken with ultrasound. The whole process was performed in room temperature. The samples for *in vivo* examinations were cylinder-shaped with the diameter of 3.2 mm and the length of 1.5 cm; they were produced through the injection method at the pressure of 12 MPa and temperature 180 °C. Both kinds of samples underwent plasma sterilization.

### 3.3. Cell Culture

For *in vitro* examinations evaluating the cytotoxicity of the assessed materials the researchers used hFOB 1.19 human osteoblast-like cells purchased at the American Type Culture Collection – ATCC (Manassas, VA, USA), catalogue number CRL – 11372. The examinations were conducted according to the indications of the PN-EN ISO 10993-5 norm [28].

The cells were cultured in 50 mL plastic bottles (Nunc A/S Roskilde). Dulbecco’s Modified Eagle’s Medium was used together with Ham’s F12 in proportion 1:1 (without phenol red and antibiotics) with the addition of 2.5 mM l-glutamine, 0,3 mg/mL G418 Sulphate and 10% foetal bovine serum (FBS) inactivated thermally. The cells were cultured in a continuous manner at a temperature of 34°C, air containing 5% CO_2_, and 100% relative humidity. The cells used in the research were after six passages, which guaranteed their stability and a constant rate of proliferation.

The samples (discs) of the materials examined (PLGA, PLGA+CF and PLGA+HA) were placed at the bottom of the pits of the four–pit cell cultivation plate containing 1 mL of the cultivation medium; next, the materials were contacted with a one-layer culture of human osteoblasts. The plates were incubated at the temperature of 37 °C in the air containing 5% CO_2_ at 100% relative humidity. The incubation time was 24, 48 and 72 hours.

### 3.4. LDH (Lactate Dehydrogenase) Release Assay

Lactate dehydrogenase is released from the cytoplasm into the culture medium as a result of damage to the cellular membrane and cellular lysis. Increased LDH activity in the supernatants of cell cultures correlates with the percentage of dead cells. LDH activity was measured using a commercial cytotoxicity assay kit (Roche Diagnostics GmbH, Mannheim, Germany), in which released LDH in culture supernatants is measured with a coupled enzymatic assay, resulting in conversion of a tetrazolium salt into red formazan product. The marking of LDH activity was done in 96-pit plates according to the procedure indicated by the manufacturer [29]. The toxicity percentage CT [%] was calculated according to formula (1), to which the values of particular absorbances were inserted, after having subtracted the values the absorbance values of the culture medium:

(1) $$CT[\%]=\left[\frac{A_{s}-A_{lc}}{A_{hc}-A_{lc}}\right]\times 100\%$$

where A_s_ – absorbance of the examined sample, A_hc_ – high control absorbance, or the value of the total LDH release (maximum LDH release after adding 1% Triton X-100 solution to cell culture), A_lc_ – low control absorbance, or the value of spontaneous LDH release (spontaneous LDH release during native cells cultivation).

### 3.5. In Vivo Experiment

Experimental research in *in vivo* conditions was performed on 84 mixed-breed rabbits of both sexes weighing 2,600 – 3,200 grams. During the operation general intravenous anesthesia with Ketamine was used, after having applied Diazepam and Atropine. The animals were divided into three equal groups – each consisting of 28 animals – one group for each of the materials examined. Incisions were made at the mandible base to get access to its lateral surfaces. Bone canals of 3.2 mm diameter were made on both sides of the animals’ mandibles with the use of a cutter fixed at the turbine of a clinical drill. Previously-prepared cylinders made of PLGA co-polymer and its composites (PLGA+CF and PLGA+HA) were inserted into the right-side canals. The left-side bone canals were filled with blood clot (control group). The wounds in the submandible area were layer-sutured with Dexon. After sacrificing the animals on the 7^th^, 14^th^ and 21^st^ day and in the 6^th^, 12^th^, 24^th^ and 48^th^ week of the experiment, histopathological and immunohistochemical evaluation of the preparations sampled was made.

### 3.6. Histopathological and Immunohistochemical Examination

The tissue specimens to histopathological and immunohistochemical examinations were preserved in 10% solution of neutralized formalin. The osseous tissue was decalcified in 10% solution of disodium versenate or electrolytically in Romeis liquid (80 mL of hydrochloric acid + 100 mL of formic acid made up to 1000 mL with water) using bone decalcifier PW23, at the electric current intensity of 0.5A. Next, all the tissue was brought in a typical way in Technicon’s Duo (to histopathological examinations) or Shandon Citadel 2000 (to immunohistochemical examinations) autotechnicon through the sequence of 96% alcohol, acetone and xylene, and then embedded in paraplast. The cubes thus obtained were then shaved on a Microm HM335E rotating microtome. The shavings of 4–6 micron thickness were put on basic slides, deparafinized and they underwent routine staining with hematoxylin-eosin (H.E.).

All the preparations to histopathological examinations were then examined with the use of Olympus BX50 light microscope with the add-on device for taking optical microphotographs - Olympus SC35 and digital ones - Olympus Camedia C-5050 Zoom; the magnification used was 40 – 400 times. Apart from that, they were consulted with the use of Nikon Labophot-2 microscope with a binocular head. The state of the bone tissue and the bone marrow around the implants canal was assessed, as well as the degradation of the cylinder made of the PLGA and its composites. Also, the rate of tissue healing was assessed, as well as the response of the organism’s hard tissue (tissue reactions) on the implant fragments penetrating them.

The shavings for immunohistochemical examinations were deparafinized in xylene, brought through the sequence of alcohols (from 100% to 96%) and then the activity of endogenous peroxidase was blocked in 3% solution of hydrogen peroxide H_2_O_2_. All the preparations were incubated for 10 minutes with equine serum. Next, they were incubated for 60 minutes with the first anti-body (against osteopontin – Osteopontin NCL-O-PONTIN and osteonectin – Osteonectin NCL-O-NECTIN respectively) in room temperature. After the first incubation, the next one was made in room temperature with the second biotynylated anti-body from Dako Company LSAB2 System-HRP kit. Another step was the incubation with Streptavidin-HRP complex fom LSAB2 System-HRP kit. The preparations were tinted with the use of chromogen (DAB – 2-aminobenzene) and the cell nuclei were additionally tinted with Mayer hematoxylin. The final stage was to bring the preparations through a reverse sequence of alcohols (from 96% to 100%) and in xylene. Each stage of immunohistochemical examinations preparation was preceded by rinsing the preparations in a buffer (TBS or PBS respectively). The preparations were evaluated in a light microscope at 400 times magnification. During the microscopic analysis the assessment used was the semiquantitative analysis of positive immunohistochemical reactions based on the percentage of the colored cells (osteoblasts, odontoblasts) and the surface of the colored extracellular matrix of the new bone tissue. The progress of the reaction was assessed after the 1^st^, 2^nd^, 3^rd^ and 6^th^ week of the experiment.

### 3.7. Statistical Analysis

The results of LDH release assay are expressed as mean values of cytoxicity CT [%] ± standard deviation (SD) from three separate repetitions. Variables distribution was evaluated by the Shapiro-Wilk test. Variation homogeneity was assessed by the Levene test. ANOVA for repeated measurements with contrast analysis were done to assess time and copolymer type interaction. To check sphericity Mauchley test was done. Differences were considered to be statistically significant at *p<0.05.* All calculations were performed using the commercially available statistical package Statistica 8.0.

## 4. Conclusions

The *in vitro* and *in vivo* examinations carried out during this research demonstrated that PLGA copolymer as well as its composites PLGA+CF and PLGA+HA are fully biocompatible materials. Their presence allows for full formation of proper bone tissue; moreover, they do not have any toxic influence upon bone-forming cells. The time and the way the materials become decomposed harmonizes with the bone regeneration process and the kind of the phase modifying the PLGA copolymer warp influences mainly the dynamics of new bone tissue formation. The conclusions drawn from the present work can be a basis for further research using the same materials in humans.

## Figures and Tables

**Table 1.** LDH activity in supernatants of human osteoblasts cultivation expressed as the percentage of cells which underwent lysis (CT) after incubation for 24, 48 and 78 hours directly with PLGA co-polymer and its composite with carbon fibers (PLGA+CF) or hydroxyapatite (PLGA+HA).

**CT [%] – LDH test**						
**Time**		**24 h**	**48 h**	**72 h**	**p_24–48_**	**p_48–72_**
1	**PLGA**	3.4±2.7	4.8±4.1	1.1±0.9	NS	0.1
2	**PLGA+CF**	2.5±0.6	8.4±2.9	0.7±0.7	**<0.01**	**<0.01**
3	**PLGA+HA**	10.4±1.6	7.0±2.4	0.6±1.0	**<0.05**	**<0.05**
	**p_1–2_**	**<0.01**	NS	NS		
	**p_1–3_**	**<0.01**	NS	NS		

Mauchley sphericity test: statistically not significant.

**Table 2.** The intensity of immunohistochemical reactions by using monoclonal antibodies to osteonectin and osteopontin.

**Osteonectin / osteopontin activity**					
**Period**		**1^st^ week**	**2^nd^ week**	**3^th^ week**	**6^th^ week**
1	**PLGA**	(++) / (++)	(+) / (+)	(+) / (+)	(+) / (+)
2	**PLGA+CF**	(+−) / (+−)	(+−) / (+−)	(+−) / (+−)	(+−) / (+−)
3	**PLGA+HA**	(++) / (++)	(++) / (++)	(+) / (+)	(+) / (+)

(+−) – 10% – 33% of tinted cells or matrix – weak reaction.

(+) – 33% – 66% of tinted cells or matrix – medium reaction.

(++) – 66% – 100% of tinted cells or matrix – strong reaction.
